# Supplementary material for: Identification of KKL-35 as a Novel Carnosine Dipeptidase 2 (CNDP2) Inhibitor by In Silico Screening
Source: Molecules. 2025 Nov 12;30(22):4370. doi: 10.3390/molecules30224370 (PMC12655758; doi:10.3390/molecules30224370)
Supplement: Supplementary file 1 [file molecules-30-04370-s001.zip › molecules-3931825-supplementary.pdf]

Figure S1

A

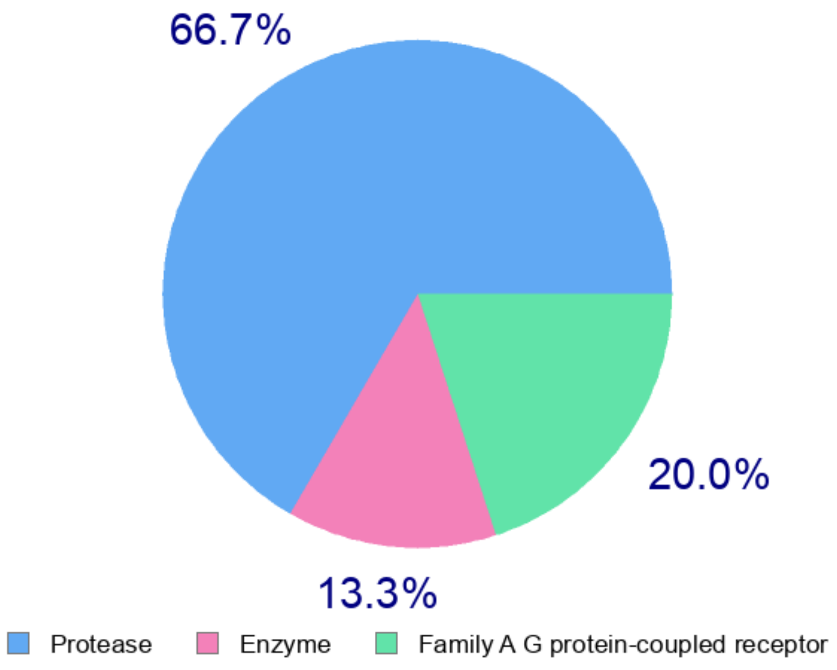

B

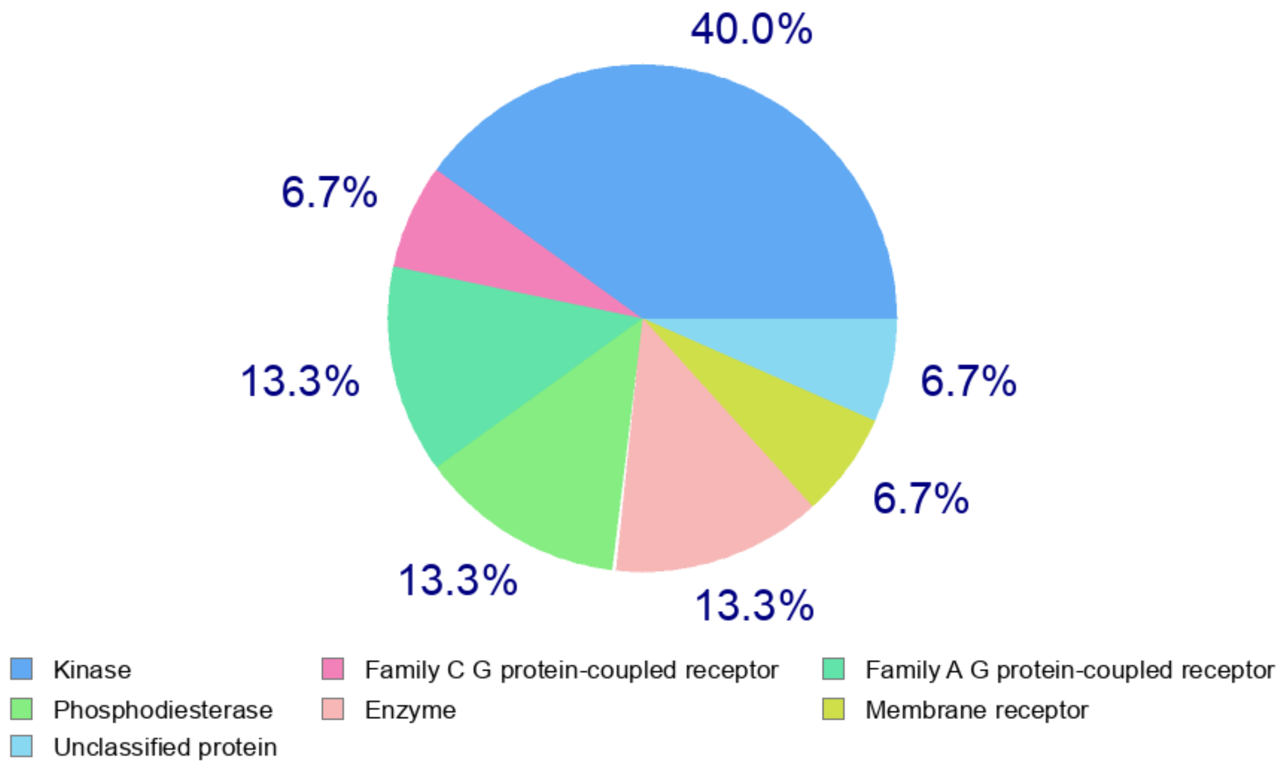

Figure S1. Predicted target classes for BES and KKL-35.
Pie charts from SwissTargetPrediction show that (A) BES is mainly predicted to target proteases (66.7%), whereas (B) KKL-35 is predicted to interact with more diverse classes including kinases, phosphodiesterases, enzymes, and G protein-coupled receptors.
